# The solution structure of the unbound IgG Fc receptor CD64 resembles its crystal structure: Implications for function

**DOI:** 10.1371/journal.pone.0288351

**Published:** 2023-09-21

**Authors:** Gar Kay Hui, Xin Gao, Jayesh Gor, Jinghua Lu, Peter D. Sun, Stephen J. Perkins

**Affiliations:** 1 Department of Structural and Molecular Biology, Darwin Building, University College London, London, United Kingdom; 2 Structural Immunology Section, Laboratory of Immunogenetics, National Institute of Allergy and Infectious Diseases, National Institute of Health, Rockville, Maryland, United States of America; University of Nova Gorica, SLOVENIA

## Abstract

FcγRI (CD64) is the only high-affinity Fcγ receptor found on monocytes, macrophages, eosinophils, neutrophils and dendritic cells. It binds immunoglobulin G (IgG) antibody-antigen complexes at its Fc region to trigger key immune responses. CD64 contains three immunoglobulin-fold extracellular domains (D1, D2 and D3) and a membrane-spanning region. Despite the importance of CD64, no solution structure for this is known to date. To investigate this, we used analytical ultracentrifugation, small-angle X-ray scattering, and atomistic modelling. Analytical ultracentrifugation revealed that CD64 was monomeric with a sedimentation coefficient *s*^*0*^_20,*w*_ of 2.53 S, together with some dimer. Small-angle X-ray scattering showed that its radius of gyration *R*_*G*_ was 3.3–3.4 nm and increased at higher concentrations to indicate low dimerization. Monte Carlo modelling implemented in the SASSIE-web package generated 279,162 physically-realistic trial CD64 structures. From these, the scattering best-fit models at the lowest measured concentrations that minimised dimers revealed that the D1, D2 and D3 domains were structurally similar to those seen in three CD64 crystal structures, but showed previously unreported flexibility between D1, D2 and D3. Despite the limitations of the scattering data, the superimposition of the CD64 solution structures onto crystal structures of the IgG Fc-CD64 complex showed that the CD64 domains do not sterically clash with the IgG Fc region, i.e. the solution structure of CD64 was sufficiently compact to allow IgG to bind to its high-affinity Fcγ receptor. This improved understanding may result in novel approaches to inhibit CD64 function, and opens the way for the solution study of the full-length CD64-IgG complex.

## Introduction

The human immune system contains five classes of immunoglobulins, of which IgG is the most abundant. IgG antibodies bind to foreign antigens such as pathogens and viruses that have entered the body to form an antigen-antibody immune complex. The immune response is activated through the binding of these immune complexes to key Fcγ receptors. There are three classes of membrane-bound human Fcγ receptors (FcγR), namely FcγRI (CD64), FcγRIIA/B/C (CD32A/B/C) and FcγRIIIA/B (CD16A/B). The three FcγR classes exhibit different affinities against the four IgG subclasses, namely IgG1, IgG2, IgG3 and IgG4. FcγRI, termed CD64 hereafter, is the only high-affinity FcγR (dissociation constant *K*_*D*_ of ~10^−8^ M) and binds free or monomeric IgG1, IgG3 and IgG4, thus FcγRI sites are occupied *in vivo*. The two classes of low affinity receptors FcγRIIA/B/C and FcγRIIIA/B (*K*_*D*_ ~10^−5^ to 10^−7^ M) bind to IgG found in antigen-antibody immune complexes, and these receptor binding sites are assumed to be unoccupied *in vivo* and available for IgG-dependent cellular reactions [1, 2]. The FcγRII and FcγRIII *K*_*D*_ values are 23 μM and 123 μM for IgG1 and IgG4 respectively [3]. CD64 functions as an activating FcγR via a cytoplasmic immunoreceptor tyrosine-based activation motif, which results in immune effector functions such as phagocytosis, antigen presentation, antibody-dependent cellular cytotoxicity and mediator secretion [4]. The molecular role of CD64 in immunity and the clearance of preformed immune complexes is still unclear [5]. It has been implicated with diseases such as arthritis, systemic lupus erythematosus and inflammatory bowel disease [6–8]. For example, CD64 is upregulated in patients with arthritis and FcγR functional activity is altered [9, 10].

**Fig 1:**
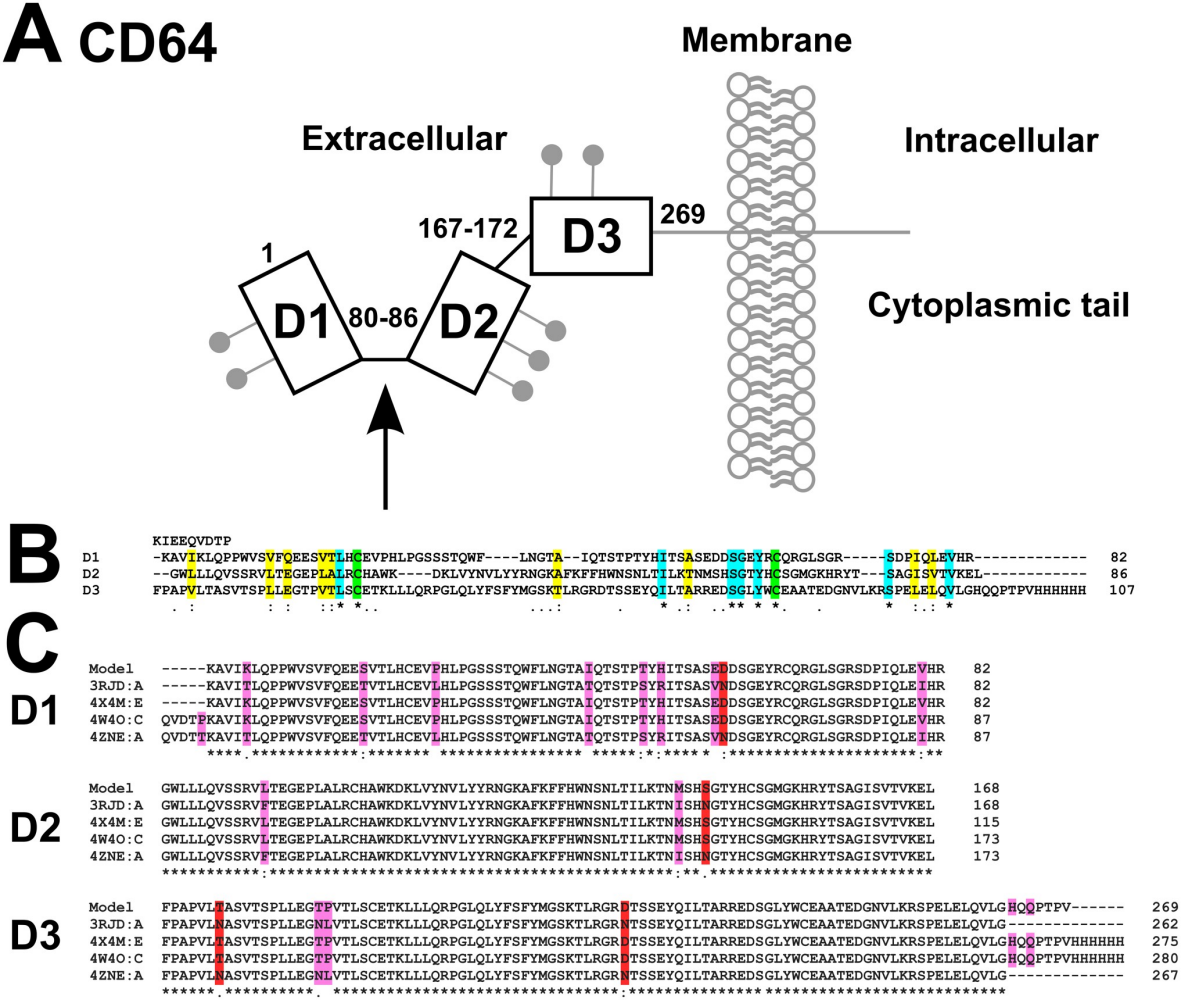
The human FcγRI (CD64) domain structure and sequences. (A) The three extracellular D1, D2, D3 domains are denoted by boxes, with seven potential N-linked glycosylation sites (●). The receptor is membrane-anchored with a short cytoplasmic tail. The black arrow indicates the binding site for the formation of the IgG-CD64 complex. Residues 80–86 and 167–172 were varied in the torsion angle Monte Carlo modelling. (B) The D1, D2 and D3 sequences were aligned using Clustal Omega. The residue lengths here and below are shown on the right. Fully conserved residues are asterisked (*; cyan), conserved cysteines are shown in green, strong similarities are shown by a colon (:; yellow), and weak similarities are shown by a period (.). The sequence of recombinant CD64 studied here is shown, starting from KIERQV and ending in HHHHHH. (C) The modelled CD64 sequence was aligned with the sequences of CD64 in its crystal structures (PDB codes: 3RJD, 4X4M, 4W4O and 4ZNE). The residues that were mutated in recombinant CD64 are highlighted in pink and red (red denoting the removal of N residues at four glycosylation sites). Fully conserved residues are asterisked (*), strong similarities are shown by a colon (:), and weak similarities are shown by a period (.).

CD64 is a 72 kDa transmembrane glycoprotein expressed on cells such as monocytes, macrophages, eosinophils, neutrophils and dendritic cells [2, 11, 12]. Human CD64 is composed of a signal sequence (residues 1–15), three extracellular immunoglobulin-like domains, D1, D2 and D3 (residues 16–292), a transmembrane region (residues 293–313) and a short cytoplasmic tail (residues 314–374 (Fig 1A and 1B) (UniProt ID: P12314) [13–15]. There are seven potential glycosylation sites (residues N59, N78, N152, N159, N163, N195 and N240). CD64 is the only FcγR with three extracellular domains [13]. In CD64, D1 and D2 interact with the Fc region of IgG, with D2 participating in most of the interactions. D3 may be important to maintain receptor conformation and stability, or act as a spacer to accommodate the Fab regions [12, 13, 16]. D3 might prevent the dissociation of CD64 and the Fc region of IgG1, thus strengthening CD64-Fc complex formation [17]. One crystal structure is available for unbound CD64 [13] and three more for the complex between CD64 and the Fc region of IgG1 (Fig 1C) [12, 16, 18]. In principle, the CD64 structure can be flexible between the D1-D2 and D2-D3 domains. In fact, only D3 showed variable locations in the four crystal structures, most likely attributable to different crystal contacts [12, 16, 18]. The *N*-terminal and *C*-terminal residues in D1-D3 were also unresolved by X-ray crystallography, suggesting structural flexibility there [12, 18].

A solution structure determination for the unbound CD64 domains will clarify its functional role. Soluble CD64 without glycans had previously been purified from *E*. *coli* [16, 17, 19, 20]. The 19 mutations in this recombinant CD64 have little effect on its structure and interaction with the Fc region (Fig 1C) [12, 18]. Human FcγRIIIB (CD16B) has been studied by neutron scattering [21]. Analytical ultracentrifugation (AUC) and small-angle X-ray solution scattering (SAXS) are complementary solution structural methods [22–24]. A solution structure allows the use of physiological concentrations and buffers, and avoids the use of crystallisation buffers and potential crystallographic packing artefacts. The most recent SAXS technology enables automated sample handling and data analysis at even lower protein concentrations, and interestingly these proved indispensable here for CD64 [25–27]. The atomistic modelling of scattering curves is a powerful new tool for structural studies. As part of the Computational Collaborative Project in Small Angle Scattering (CCP-SAS), the SASSIE package generates physically-realistic trial atomistic models of CD64 using Monte Carlo methods [28, 29]. These models were used to generate theoretical scattering curves using an all-atom expression [30] for comparison with the experimental SAXS curves to identify best-fit solutions. Here, we show that the CD64 solution structure is mostly similar to its compact structure seen by crystallography, but we identify previously-uncharacterised conformational differences attributed to protein flexibility, and discuss the functional significance of this outcome.

## Materials & methods

### Expression and purification of recombinant CD64

The pet26b plasmid containing recombinant human CD64 (Uniprot Accession Code P12314, FCGR1_HUMAN), but with 19 mutations (Fig 1B) [16, 20], was transformed into competent *E*. *coli* (Arctic Express DE3 RIL competent cells, Agilent Technologies). Residues 16–289 (Fig 1B) that also included an N-terminal peptide KIEEQVDTP that is believed to increase secretion to the periplasm and a C-terminal His-tag, shows a sequence-calculated mass of 32,012 Da. Colonies were inoculated into 200 ml of 2×YT kanamycin (100 μg/ml) medium and grown overnight at 30 ºC with shaking at 220 r.p.m. The starter culture at an OD_600_ of 45 was inoculated into fresh 6 × 1.5 l of 2×YT kanamycin (100 μg/ml) medium and incubated at 20ºC until the OD_600_ reached 0.2. This was induced with IPTG at a final concentration of 50 μM, and incubated with shaking at 20 ºC overnight for up to 20 h. Typically 9 l were harvested at 4000 r.p.m. at 4 ºC for 20 min, the medium was discarded, and the *E*. *coli* pellets were stored at -80 ºC.

To purify CD64, the pellets were resuspended using 500 ml of resuspension buffer (30 mM Tris-HCl, 150 mM NaCl, 10 mM imidazole, pH 7.6) supplemented with 500 μl phenylmethylsulfonyl fluoride. The cells were sonicated for 10 min with 10 sec pulse on and 20 sec pulse off. The lysate was centrifuged at 14,000 r.p.m. at 8ºC for at least 30 min. The CD64 supernatant was filtered using 0.8 μm filter paper and stored at 4 ºC. An IgG Fast Flow (FF) Sepharose column was equilibrated with Dulbecco’s phosphate buffer saline (PBS; 137 mM NaCl, 8.1 mM Na_2_HPO_4_, 2.7 mM KCl, 1.5 mM KH_2_PO_4_, pH 7.4). The CD64 supernatant was loaded onto the column overnight at 4ºC, and the column was washed with PBS until a stable UV baseline was achieved. CD64 was eluted using elution buffer (0.1 M glycine, pH 2) and immediately neutralised using 100 mM Tris-HCl, 100 mM NaCl, 100 mM imidazole, pH 8. The eluent pH was checked before the purified CD64 was stored at 4ºC, and the column pH was restored using PBS. Batches were snap-frozen in liquid N_2_ and stored at -80ºC until required.

Prior to experiments, CD64 was thawed overnight at 4°C and loaded onto an IgG FF Sepharose column equilibrated with 30 mM Tris, 150 mM NaCl, 10 mM imidazole, pH 7.6. CD64 was eluted using 0.1 M glycine at pH 2 as 500 μl aliquots directly into 500 μl of neutralisation buffer (100 mM Tris, 100 mM NaCl, 100 mM imidazole at pH 8) per fraction. The fractions were checked immediately using SDS-PAGE. CD64 was concentrated at 4°C using spin concentrators pre-soaked with 30 mM Tris, 150 mM NaCl, 100 mM imidazole, pH 7.6 (Vivaspin, 10 kDa molecular weight cut-off) to a volume of 500–1000 μl. Dialysis was performed overnight into its respective buffer at 4°C using three buffer exchanges of 1 l each prior to experiments.

### Characterization of CD64

The CD64 purity was verified by Superose 6 gel filtration, SDS-PAGE, and mass spectrometry. After dialysis into 100 mM ammonium acetate, CD64 was analysed on an Agilent 6510 Quadrupole time-of-flight liquid chromatography mass spectrometry system (Agilent, UK). Ten μL of each sample was injected onto a PLRP-S, 1000A, 8 μM, 150 mm × 2.1 mm column, which was maintained at 60°C at a flow of 0.3 ml/min. Separation was achieved using mobile phases A (water with 0.1% formic acid) and B (acetonitrile, with 0.1% formic acid) using a gradient elution. The column effluent was continuously electrosprayed into the capillary electrospray ionization source of the mass spectrometer and electrospray ionization mass spectra were acquired in positive electrospray ionisation mode using the *m/z* range 1,000−3200 in profile mode. The raw data was converted to zero charge mass spectra using the maximum entropy deconvolution algorithm in the MassHunter software version B.07.00 (Agilent, Stockport, UK).

### Analytical ultracentrifugation of CD64

AUC data using both absorbance and interference optics were obtained on two Beckman XL-I instruments equipped with AnTi50 (201,600 × g at the cell bottom at 50,000 rpm) and AnTi60 rotors (290,000 × g at the cell bottom at 60,000 rpm). Sedimentation velocity data were acquired for CD64 at 20°C in either 30 mM Tris, 150 mM NaCl, pH 7.6 or 30 mM Tris, 150 mM NaCl, 100 mM imidazole, pH 7.6 buffers. CD64 was studied at 0.05–0.90 mg/ml at rotor speeds of 40,000 rpm and 50,000 rpm in two-sector cells with column heights of 12 mm. Analyses were performed using direct boundary Lamm fits of up to 350 scans using SEDFIT (version 15.1) [31, 32]. SEDFIT gave size-distribution analyses *c*(*s*) that revealed the sedimentating species; SEDFIT assumed these species to have the same frictional ratio *f/f*_0_. The final SEDFIT analyses used a fixed resolution of 200 and optimized the *c*(*s*) fit by floating *f/f*_0_ and the baseline until the overall root-mean-square deviations and visual appearance of the fits were satisfactory. The sedimentation coefficients normalised to 20°C in water were reported as *s*_*20*,*w*_ values. The partial specific volume of CD64 was calculated as 0.7363 ml/g from its sequence using SLUV [33]. The buffer density and viscosity of 1.00729 g/cm^3^ and 0.010835 poise respectively were measured for 30 mM Tris, 150 mM NaCl, 100 mM imidazole, pH 7.6 using a DMA 5000 density meter and an AMVn Automated Microviscometer (Anton Paar). The buffer density and viscosity of 1.0053 g/cm^3^ and 0.0102465 poise respectively were calculated for 30 mM Tris, 150 mM NaCl, pH 7.6 buffer using SEDNTERP version 1.09 [34].

### X-ray scattering of CD64

After 20 h dialysis into 30 mM Tris, 150 mM NaCl, pH 7.6 at 4°C, SAXS data on the CD64 samples were measured on Instrument B21, Diamond Light Source, Didcot, Oxon., UK [35]. The beamsize was 250 × 250 μm and the beam had an energy of 12.4 keV at the detector. Concentrations of 0.11, 0.22, 0.33 and 0.44 mg/ml were measured in triplicate. Sample volumes of 40 μl was loaded into each well of a 96-well plate which was sealed with a plate cover. The 96-well plate was loaded onto the BIOSAXS automatic sample changer [36]. Biosaxs Customised Beamline Environment (BsxCUBE) software was used to control the automatic sample changer, in which 30 μl volumes were pipetted automatically into a temperature-controlled quartz cell capillary for automated X-ray data collection [37]. Thirty frames of data, each for 1 sec, were recorded per sample, during which the sample was continuously moved through the capillary at a constant temperature of 20°C. The scattering intensities *I*(*Q*) were recorded on a Pilatus 2M camera at a sample-detector distance of 4 m to give a *Q*-range of 0.032–3.8 nm^-1^ (where *Q* = 4π sin θ/λ; 2*θ* is the scattering angle and *λ* is the wavelength).

For a given solute–solvent contrast, the radius of gyration *R*_*G*_ is a measure of structural elongation. Guinier analyses at low *Q* gives the *R*_*G*_ and the forward scattering at zero angle *I*(0) [38]:

$$\text{ln}\;I\left(Q\right)=\text{ln}\;I\left(0\right)-\frac{{R_{G}}^{2}Q^{2}}{3}$$

This expression is valid in a *Q*.*R*_*G*_ range up to 1.5. If the structure is elongated, the mean radius of gyration of cross-sectional structure *R*_*XS*_ and the mean cross-sectional intensity at zero angle [*I*(*Q*)*Q*]_*Q*→0_ is obtained from:

$$\text{ln}\;\left[I\left(Q\right)Q\right]={\left[I\left(Q\right)Q\right]}_{Q⟶0}-\;\;\frac{{R_{XS}}^{2}Q^{2}}{2}$$

The *I*(0) values normalised by the concentration c is proportional to the molecular mass [39]. The subtraction of the solute and buffer scattering curves was performed using ScÅtter version 3.1 (http://www.bioisis.net/tutorial/9). The *R*_*G*_ and *R*_*XS*_ analyses were performed using the SCT suite [33]. Dimensionless Kratky plots of (*Q*.*R*_*G*_)^2^.*I*(*Q*)*/I*(0) vs *Q*.*R*_*G*_ were calculated using the Guinier *R*_*G*_ values and provides information on the flexibility of the protein [40–43].

Indirect Fourier transformation of the scattering data *I*(*Q*) to give the distance distribution function *P*(*r*) was performed using the program GNOM [44]:

$$P\left(r\right)=\frac{1}{{2\pi }^{2\;}}\;\int_{0}^{\infty }I\left(Q\right)Qr\;\text{sin}\;\left(Qr\right)dQ$$

*P*(*r*) corresponds to the distribution of distances *r* between volume elements. This provides the maximum dimension *L* of CD64 and its most commonly occurring distance vector *M* in real space. For this, the *I*(*Q*) curve utilized up to 598 data points in the *Q* range between 0.18 to 1.50 nm^-1^.

### Atomistic SAXS and AUC modelling of CD64

The starting CD64 structure for simulations was generated from the crystal structure of the CD64-Fc complex (chain E; PDB code: 4X4M), this being the same recombinant CD64 as that used for the AUC and SAXS data [16]. The sequence alignment of the D1, D2 and D3 domains was performed with Clustal Omega (Fig 1B and 1C) [45]. The missing D3 loop residues ^220^RPG^222^ and C-terminal residues ^283^HQQPTPV^289^ were inserted using Modeller version 9.14 [46]. The N-terminal peptide and the Histag residues were not included for reason of their uncertain positioning. Charmm-GUI was used to prepare the structure files with force field parameterizations and hydrogen atoms. The original sequence numbering from ^21^K to ^289^V was renumbered as ^1^K to ^269^V in this study (Fig 1) [47]. An energy minimisation of 1000 steps gave the CD64 starting model using NAMD version 2.9, the Generalized Born implicit solvent model, and the CHARMM 36 forcefield [48–50]. All three disulphide bonds at Cys23–Cys65, Cys104–Cys148 and Cys192-Cys240 were retained, and hydrogen atoms were added.

Thirteen Monte Carlo simulations to generate 2,450,000 trial CD64 models were performed using the ‘Torsion Angle Monte Carlo’ module in SASSIE [28, 29]. In these, the linker residues between the D1-D2 and D2-D3 pairs ^80^VHRGWLL^86^ and ^168^LFPA^171^ were varied. The D1 and D3 domains were thus moved relative to the fixed D2 domain. Models showing steric overlap were automatically discarded, leaving 279,162 acceptable models that were merged into a single trajectory file (Table 1). The simulations used a temperature of 300 K, with maximum angles of up to 30° or 180° for each move or step (Table 1).

**Table 1:** Summary of the modelling simulations for CD64. Details of the thirteen CD64 simulations using the torsion angle Monte Carlo module in SASSIE-web are shown.

Simulation	Flexible regions (inclusive residue numbering)	Maximum torsion angle (º)	Total models generated	Accepted models
1	^82^RGW^84^, ^168^LFPA^171^	30	100,000	5,779
2	^82^RGW^84^, ^168^LFPA^171^	30	100,000	7,159
3	^82^RGW^84^, ^168^LFPA^171^	30	100,000	5,435
6	^82^RGW^84^, ^168^LFPA^171^	30	500,000	83,022
4	^82^RGW^84^, ^168^LFPA^171^	180	500,000	38,155
5	^82^RGW^84^, ^168^LFPA^171^	180	200,000	25,831
7	^82^RGW^84^, ^168^LFPA^171^	180	100,000	12,929
8	^82^RGW^84^, ^168^LFPA^171^	180	100,000	13,187
9	^82^RGW^84^, ^168^LFPA^171^	180	150,000	11,265
10	^82^RGWL^85^, ^168^LFPA^171^	180	200,000	24,912
11	^82^RGWL^85^, ^168^LFPA^171^	180	200,000	25,066
12	^80^VHRGWLL^86^, ^168^LFPA^171^	30	100,000	19,262
13	^80^VHRGWLL^86^, ^168^LFPA^171^	180	100,000	7,160

Scattering curves *I(Q)* were calculated using the ‘SasCalc’ module in SASSIE. The modelled *P(r)* curves were calculated from these using GNOM [44]. The converged number of golden vectors for a complete scattering profile was 33 using a tolerance of 0.01, at which a negligible difference was observed between the calculated scattering curves [30]. A scattering curve for each of the 279,162 models was calculated [30]. A total of 454 *I(Q)* values in a *Q*-range from 0 to 1.00113 nm^-1^ and a *Q*-spacing (Δ*Q*) of 0.002206 nm^-1^ were used. The *I*(0) values were scaled to 1. Each experimental scattering curve was interpolated using a MATLAB script (version 2013a) using the same *Q*, *Q*-range, and Δ*Q* values and scaled to *I*(0) = 1. The difference between the modelled *I*_*model*_(*Q*) and experimental *I*_*exp*_(*Q*) curves was analysed using the *R-factor* which is analogous to that used in crystallography [33]:

$$Rfactor=\sum \frac{I_{exp}\left(Q\right)-\;I_{model}\left(Q\right)}{I_{exp}\left(Q\right)}$$

This used the ‘Chi-Square Filter’ module in SASSIE [28, 29]. The 100 best-fit models with the lowest *R-factors* were identified for each scattering curve at 0.11, 0.22, 0.33 and 0.44 mg/ml. Energy minimisation (2000 steps) of each best-fit model was performed on NAMD at the flexible linkers only. Any broken models or with physically unrealistic linker conformations were discarded. Density plots were generated in SASSIE to visualise residues 1–79, 87–166 and 172–269 in terms of the rigid D1, D2 and D3 regions, and excluding the flexible regions. The density plots, DCD trajectory files and PDB coordinate files were visualised on Visual Molecular Dynamics (VMD) version 1.9.3 [51] and PyMOL (Schrödinger, LCC).

### AUC modelling of CD64

Sedimentation coefficients s^0^_20,w_ were calculated for the four CD64 crystal structures, the CD64 starting structure, and each set of 100 X-ray best-fit models for comparison with the experimental values using HYDROPRO version 10 [52]. HYDROPRO utilised the shell calculation of the atomic level primary model and a hydrodynamic radius of 0.29 nm for each of the elements in the primary model. SLUV was used to generate the partial specific volumes and molecular masses [33]. The individual partial specific volumes of 0.7385 cm^3^/g, 0.7359 cm^3^/g, 0.7363 cm^3^/g and 0.7379 cm^3^/g were used for the four crystal structures (PDB codes 3RJD, 4W4O, 4X4M and 4ZNE respectively), and one of 0.7376 cm^3^/g for the starting CD64 structure and the best-fit models. Molecular masses were calculated to be 29,367 Da, 31,453 Da, 30,913 Da and 29,911 Da for the four crystal structures respectively, and 30,091 Da for the starting structure of CD64 and the best-fit models.

## Results

### Purification and characterisation of CD64

**Fig 2:**
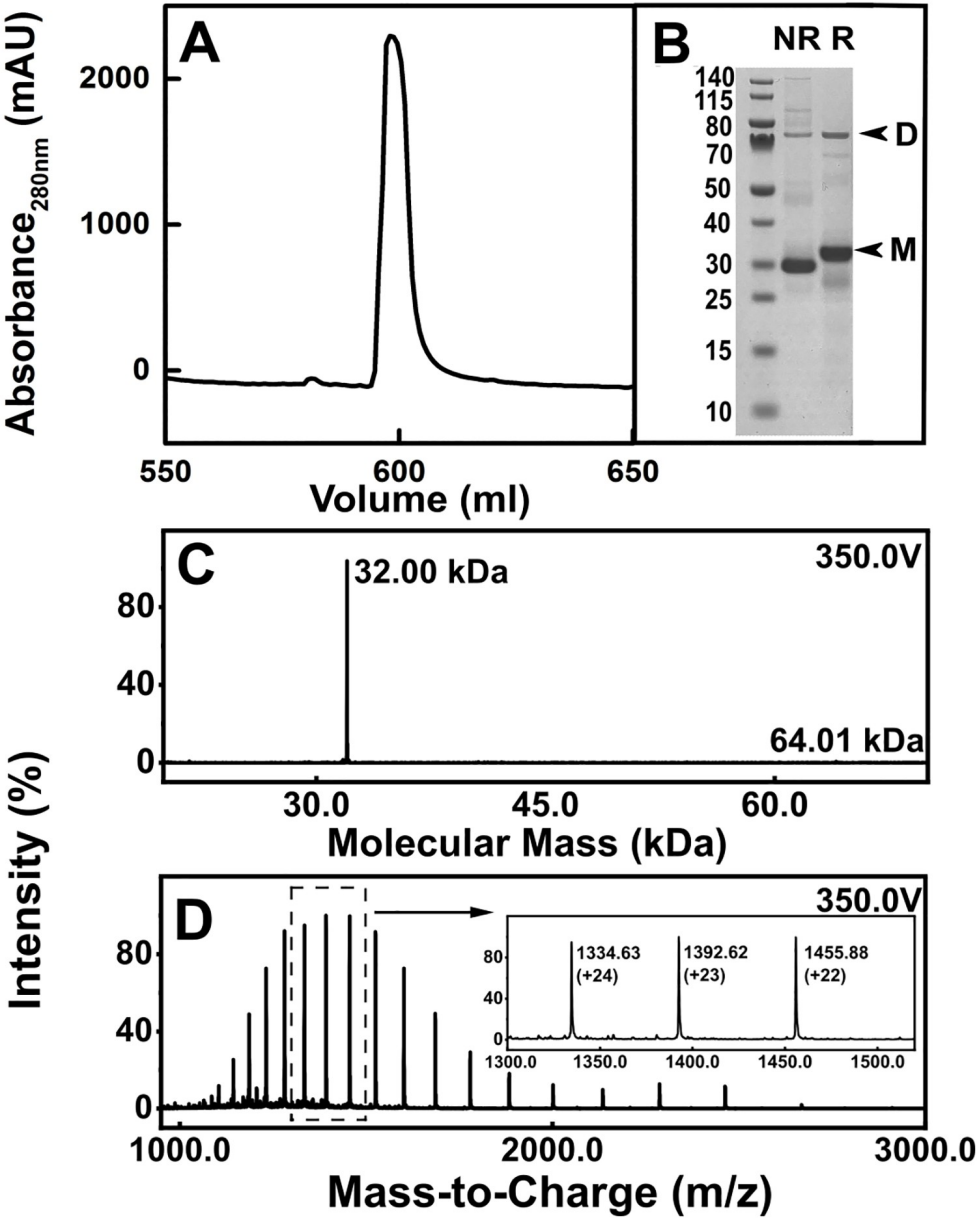
Characterisation of recombinant CD64. (A) The elution of CD64 from an IgG sepharose affinity column (mAU, milli-absorbance units) (B) SDS-PAGE analyses of CD64. Lane 2 represents the non-reduced (NR) recombinant CD64 and Lane 3 represents reduced CD64 (R). The original SDS-PAGE image is shown in S1 Fig. Most of the CD64 was monomeric (arrowed as M) while small amounts of dimer CD64 are arrowed as D. (C) The LC-MS mass spectrum for recombinant CD64 is shown as a single peak at 32.00 kDa at a voltage of 350 V, together with a small peak at 64.01 kDa. (D) The theoretical charge states generated using MassHunter software are labelled. The three strongest peaks are expanded into the panel for clarity.

Purified CD64 after a freeze-thaw cycle was subjected to affinity chromatography on an IgG Sepharose FF column to ensure that the protein was folded and functional. It was eluted using 0.1 M glycine at pH 2.0 as a large main peak at approximately 600 ml into vials containing neutralisation buffer (Fig 2A). While CD64 was expressed at 20°C, its preparation for AUC and SAXS was performed at 4°C because CD64 was functionally labile between 30–37°C [14], and its thermal stability decreased with increasing temperature [17]. The 19 mutations in recombinant CD64 had little effect on its structure and interaction with the Fc region (Fig 1C) [12, 18]. The mutants had been introduced to improve the thermal stability and production rate of CD64 [19]. SDS-PAGE revealed a single band close to 31 kDa (Fig 2B) that corresponded to the expected CD64 mass of 32,012 Da, however a small dimer peak at around 75 kDa was also seen. The monomer mass agreed with that in previous SDS-PAGE for non-glycosylated CD64 [12, 17, 20]. Mass spectrometry runs in quadriplicate revealed a sharp single signal with a mass of 32.0 kDa (Fig 2C and 2D) and a small dimer signal at 64.0 kDa. CD64 was thus of high purity.

### Analytical ultracentrifugation of CD64

**Fig 3:**
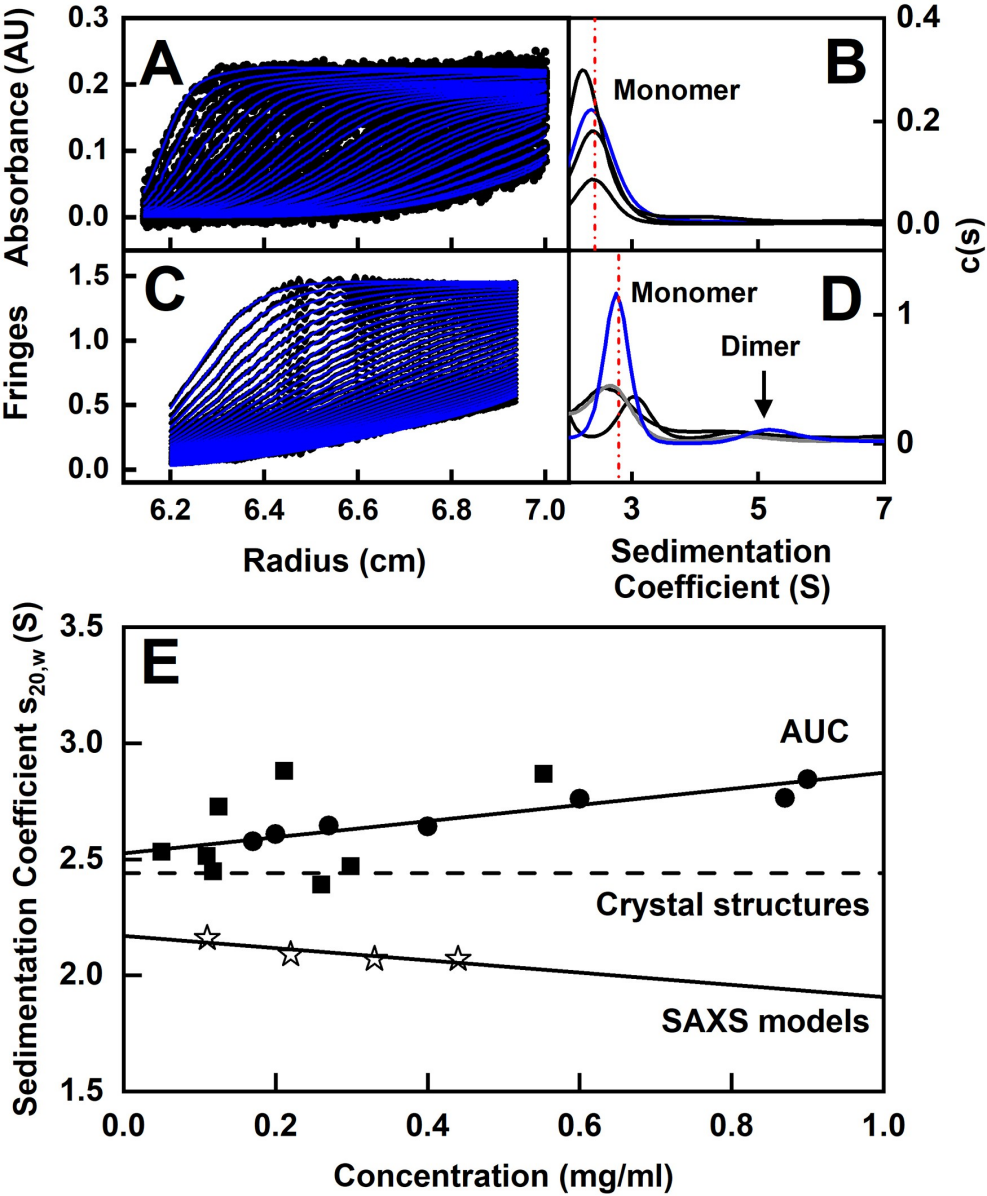
Experimental and modelled sedimentation analyses of CD64. (A-D) Sedimentation velocity analyses for CD64 in 30 mM Tris, 150 mM NaCl, pH 7.6, 20°C are shown for *A*,*B*, absorbance optics and *C*,*D*, interference optics. Up to 30 sedimentation boundaries are shown from a total of 150 scans. The boundary fits are shown in blue (left), together with the corresponding *c*(*s*) plot (right). The vertical dashed line (red) shows the average sedimentation coefficient. (E) The concentration dependence of the experimental *s*_20,*w*_ values were recorded in the above buffer (■) and also with the addition of 100 mM imidazole to this buffer (●). The solid best-fit line shows the experimental *s*_20,*w*_ values. The *s*^*0*^_20,*w*_ values from HYDROPRO are displayed as a dashed line for four known crystal structures (Methods). The *s*_20,*w*_ regression line is shown from the scattering best-fit models of CD64 (☆) at four concentrations.

The size and shape of CD64 was examined using sedimentation velocity experiments by AUC. Here and below, CD64 was measured at concentrations below 1 mg/ml for reason of its propensity to aggregate. Up to 350 scans were acquired per AUC sample, and SEDFIT analyses showed excellent agreement between the experimental boundary scans and fitted lines (Fig 3A and 3C). The size distribution analyses *c(s)* revealed a major monomer peak at ~2.5 S together with low amounts of dimer at ~5 S for CD64 seen by interference optics (Fig 3B and 3D). The monomer was observed at *s* values of 2.41 ± 0.14 S and 2.79 ± 0.20 S for the absorbance and interference data respectively (red lines, Fig 3B and 3D). Variations in these measurements were attributed to the low concentrations in use.

A total of 28 data points (19 interference and 9 absorbance) were collected for CD64 in 30 mM Tris, 150 mM NaCl, pH 7.6 buffer, and 14 data points (7 interference and 7 absorbance) were collected using 30 mM Tris, 150 mM NaCl, 100 mM imidazole, pH 7.6 buffer. The *s*_20,*w*_ values for the absorbance data showed a slight concentration dependence consistent with a fast exchange between monomer and small amounts of dimer (Fig 3E); the interference data were not included for reason of clarity. Linear regression resulted in an *s*^*0*^_20,*w*_ value at zero concentration of 2.53 ± 0.06 S, and no difference was seen between the two buffers. The CD64 shape modelling of the *s*^*0*^_20,*w*_ value is described below.

### X-ray scattering of CD64

**Fig 4:**
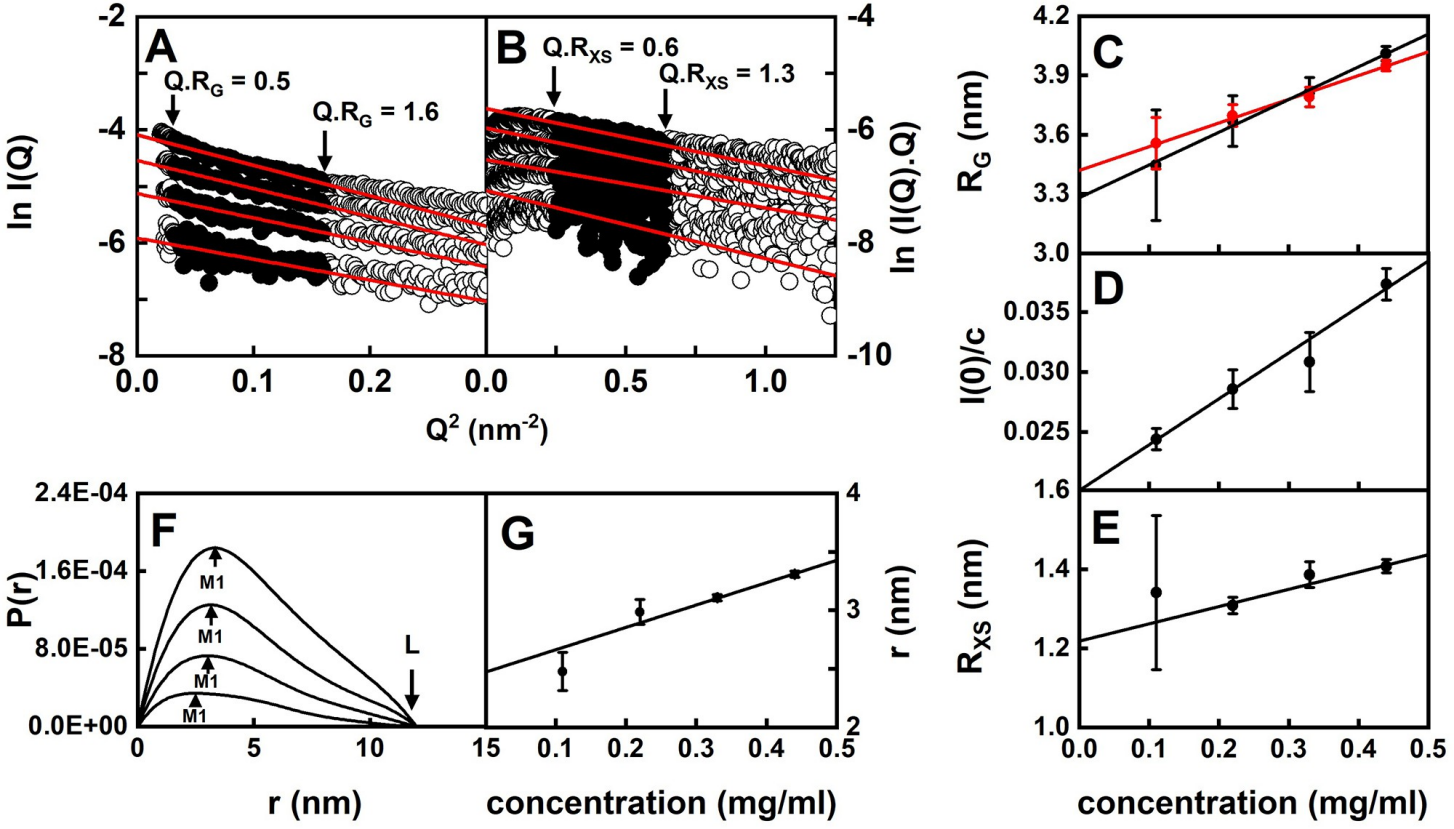
X-ray Guinier and P(r) analyses of CD64. (A,B) Representative X-ray Guinier plots at 0.11, 0.22, 0.33 and 0.44 mg/ml CD64 are shown from bottom to top, each measured in triplicate in 30 mM Tris, 150 mM NaCl, pH 7.6, 20°C. The filled circles between the arrowed data points represent the *Q*.*R*_*G*_ and *Q*.*R*_*XS*_ ranges used to determine the *R*_*G*_ and *R*_*XS*_ values, in *Q*-ranges of 0.18–0.40 nm^-1^ and 0.5–0.8 nm^-1^ respectively. (C-E) The X-ray *R*_*G*_, *I*(0)*/c* and *R*_*XS*_ values measured in triplicate are shown as the mean ± standard deviation. The Guinier values are shown in black, and the *P*(*r*) values are shown in red. The data were fitted by linear regression. (F,G) The *P*(*r*) analyses from the X-ray curves shown in *A*,*B* show maxima at *M1* and maximum lengths *L* as arrowed. The concentration dependence of *M1* fitted by linear regression is shown as their mean ± standard deviation. Error bars are shown only when visible.

Size and shape data on CD64 were obtained also by SAXS at 20°C in 30 mM Tris, 150 mM NaCl, pH 7.6 at concentrations between 0.11 and 0.44 mg/ml in triplicate. Despite signal-noise issues caused by the low concentrations in use, Guinier analyses resulted in high-quality linear plots in two distinct regions of the *I*(*Q*) curves that gave the *R*_*G*_ and *R*_*XS*_ values within satisfactory *Q*.*R*_*G*_ and *Q*.*R*_*XS*_ limits (Fig 4A and 4B). The *R*_*G*_, *I*(0)*/c* and *R*_*XS*_ values showed concentration dependences (Fig 4C–4E). The *R*_*G*_ values increased from 3.45 ± 0.28 nm to 4.01 ± 0.04 nm (Fig 4C). The *I*(0)*/c* values also increased with concentration from 0.0244 ± 0.0009 to 0.0373 ± 0.0013 which showed increased masses attributable to dimerization, in agreement with the AUC data (Fig 4D). The *R*_*XS*_ values also increased slightly with average values of 1.34 ± 0.19 nm, 1.31 ± 0.02 nm, 1.39 ± 0.03 and 1.41 ± 0.02 nm at concentrations of 0.11, 0.22, 0.33 and 0.44 mg/ml respectively (Fig 4E).

The distance distribution function *P*(*r*) provides structural information on CD64 in real space. The *P*(*r*) analyses gave *R*_*G*_ values similar to those from the Guinier analyses, showing these were self-consistent (red and black, Fig 4C). These were 3.56 ± 0.13 nm (0.11 mg/ml), 3.70 ± 0.06 nm (0.22 mg/ml), 3.79 ± 0.05 nm (0.33 mg/ml) and 3.95 ± 0.03 nm (0.44 mg/ml). The maximum length *L* of CD64 was estimated to be 12 nm from the value of *r* when the *P*(*r*) curve intersects zero (Fig 4F). The *M1* maximum in the *P*(*r*) curves correspond to the most frequently occurring interatomic distances *r*, and these increased with concentration (Fig 4G) with values of 2.48 ± 0.16 nm (0.11 mg/ml), 2.98 ± 0.11 nm (0.22 mg/ml)), 3.11 ± 0.03 nm (0.33 mg/ml) and 3.31 ± 0.03 nm (0.44 mg/ml). These increases were attributed to some dimer formation.

### Atomistic modelling of CD64

**Fig 5:**
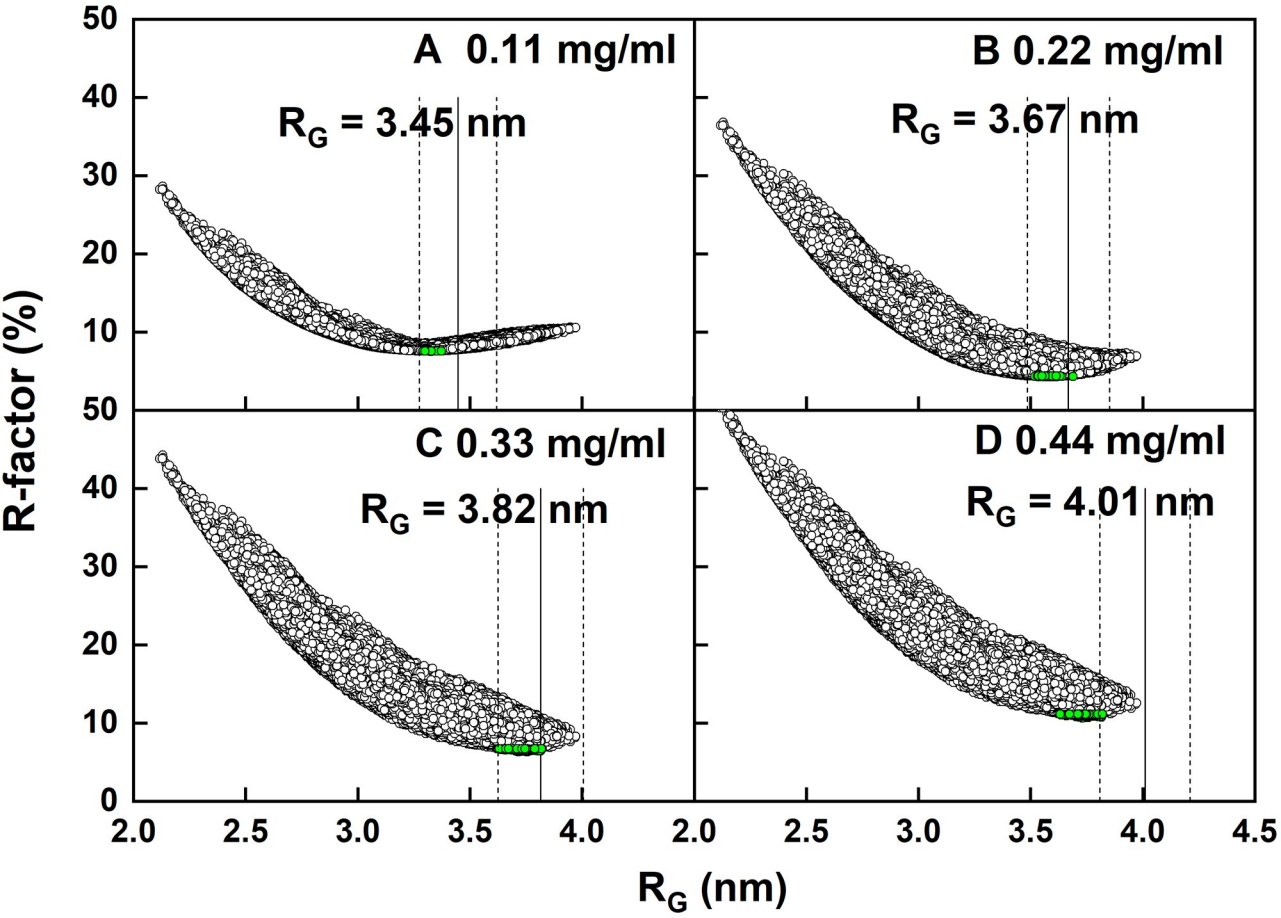
Atomistic modelling of the CD64 solution structure. The 279,162 goodness-of-fit *R*-factors (○) at four concentrations of CD64 at (A) 0.11 mg/ml, (B) 0.22 mg/ml, (C) 0.33 mg/ml and (D) 0.44 mg/ml were compared with the calculated X-ray *R*_*G*_ values for the CD64 models. The *R*-factors of the 100 best-fit models for each concentration of CD64 are shown as green circles. The experimental Guinier *R*_*G*_ values (Fig 4C) are shown as thick vertical black lines, flanked by vertical dashed lines to denote ±5% of the *R*_*G*_ value.

The atomistic modelling of CD64 to fit the SAXS data was initiated, this being focussed on the CD64 monomer. Starting from the CD64-Fc crystal structure, Modeller was used to insert ten missing residues in the D3 domain (Methods). The N-terminal nine and six C-terminal His-tag residues were not included. NAMD energy minimisation positioned the amino acid residues according to the CHARMM forcefields to generate the starting structure of CD64. The comparison of the crystal and starting structures based on 244 Cα atoms gave an RMSD of 0.0756 nm using the “align” function in PyMOL. The peptide linkers between the D1-D2 and D2-D3 domains were assigned to be flexible and their conformations were randomly varied in thirteen Monte Carlo simulations (Table 1) to generate 2,450,000 models. Of these, 279,162 models (11.4%) were stereochemically acceptable and thus used for SAXS curve fits. To explore the perturbations caused by dimer formation, the modelling fits were performed using the scattering curves at all four CD64 concentrations (Table 2). The scattering curve and its *R*_*G*_ value were calculated for each CD64 model for comparison with the experimental scattering curves using *R-factors* to monitor the goodness-of-fit. The models with the 100 lowest *R-factors* could be identified for each SAXS concentration in U-shaped profiles (Fig 5). The use of more or fewer best-fit models did not alter the outcome of the analyses. In all four cases, the 100 best-fit structures showed *R*_*G*_ values that were generally within ±5% of the observed experimental Guinier *R*_*G*_ values. From the increase in the R_G_ values (Fig 5), the increasing amounts of dimer at higher concentrations caused the best-fit CD64 monomer conformation to apparently become more elongated.

**Table 2:** Structural modelling of the SAXS and AUC data for CD64.

Concentration (mg/ml)	Sample name	Experimental Guinier *R*_*G*_ (nm)	Modelled Guinier *R*_*G*_ (nm)	*R*_*G*_ range of the 100 best-fit models* (nm)	*R-factor* range for the 100 best-fit models (%)	*R-factor* range for all models (%)	*s*_*20*,*w*_ value of best-fit model** (S)	*s*_20,*w*_ range of the 100 best-fit models** (S)
0.11	D11	3.24	3.24	3.23–3.25	8.08–8.08	8.08–27.69	2.09	2.23–2.30
	E11^a^	3.34	3.33	3.29–3.37	7.54–7.57	7.54–28.61	2.09	2.12–2.19
	25p_IF11	3.76	3.80	3.73–3.82	7.35–7.41	7.35–34.28	2.00	2.06–2.12
0.22	D10	3.59	3.58	3.52–3.69	4.05–4.20	4.05–37.19	2.00	2.05–2.11
	E10^b^	3.60	3.60	3.52–3.69	4.26–4.37	4.26–36.81	2.02	2.06–2.13
	50p_IF10	3.82	3.75	3.66–3.82	5.05–5.29	5.05–40.72	1.99	2.05–2.10
0.33	D9	3.73	3.58	3.54–3.69	9.19–9.27	9.18–37.00	2.01	2.05–2.14
	E9	3.85	3.76	3.63–3.82	6.49–6.91	6.49–44.67	1.99	1.81–2.16
	F9^c^	3.86	3.75	3.63–3.82	6.33–6.71	6.33–44.26	1.99	2.05–2.10
0.44	D8	3.97	3.73	3.63–3.82	9.75–10.24	9.75–49.48	1.99	2.05–2.10
	E8	4.04	3.73	3.63–3.82	11.12–11.61	11.12–51.42	1.99	2.05–2.10
	100p_IF8^d^	4.02	3.73	3.63–3.82	10.66–11.15	10.66–50.75	1.99	2.05–2.10

* The R_G_ range of all models is 2.12 to 3.97 nm

** Calculated from HYDROPRO and converted into *s*_20,*w*_ values

^a, b, c, d^ The corresponding data are shown in Fig 4

**Fig 6:**
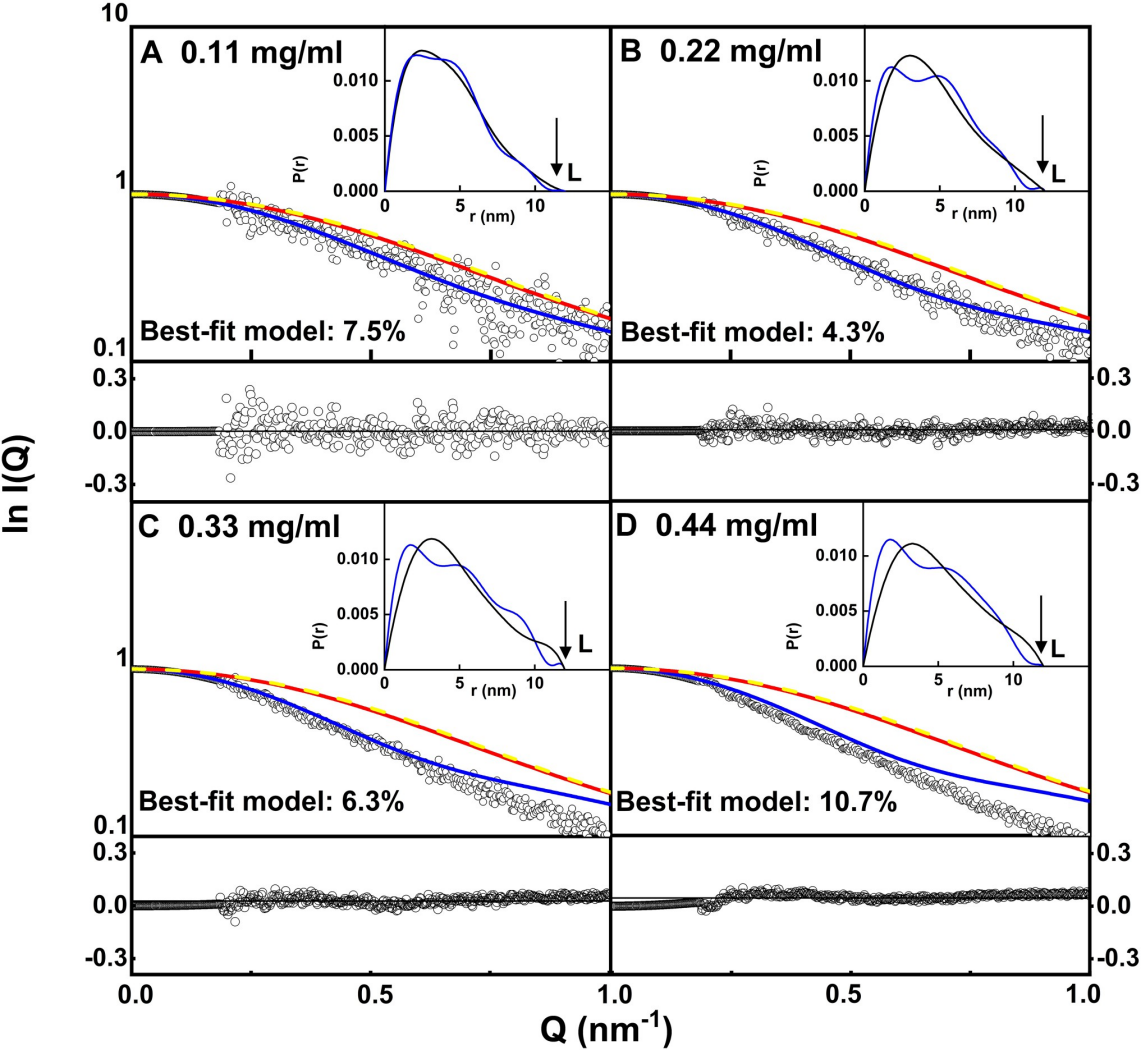
X-ray scattering curve fits for the best-fit CD64 models. The interpolated experimental X-ray scattering curves are indicated by open circles and the scattering curves of the best-fit models are indicated by blue continuous lines. The fits correspond to (A) 0.11 mg/ml, (B) 0.22 mg/ml, (C) 0.33 mg/ml and (D) 0.44 mg/ml of CD64 (Fig 4). The red lines represent the modelled curve for the energy-minimised starting structure. The yellow dashed lines represent the calculated scattering curve from the CD64 crystal structure (chain E, PDB code 4X4M). The insets represent the corresponding modelled *P*(*r*) curves (blue) overlaid onto the normalised experimental *P*(*r*) curves (black) from Fig 4F.

Visual comparisons of the best-fit *I(Q)* and *P*(*r*) curves with the experimental curves showed that the fit at 0.11 mg/ml CD64 gave a good fit to the experimental *I(Q)* and *P*(*r*) curves (blue, Fig 6A). There, the residuals are random on either side of the fitted curve. The *R-factor* of 7.5% was high because the CD64 concentration was low. The curve fit for 0.22 mg/ml CD64 to the *I(Q)* curve gave a lower *R-factor* of 4.3% (blue, Fig 6B), but showed small deviations in the *P*(*r*) fit. The fits for 0.33 mg/ml and 0.44 mg/ml CD64 showed larger deviations with the experimental *I(Q)* curve above *Q* = 0.7 nm^-1^ (blue, Fig 6C and 6D), and the *P*(*r*) fits were poorer too. These larger differences were attributed to the increased proportions of dimer. Overall, the best agreement with the yellow and red curves (crystal and modelled structures respectively) was obtained with the 0.11 mg/ml curve, in which the amount of dimer was the lowest (Fig 6).

**Fig 7:**
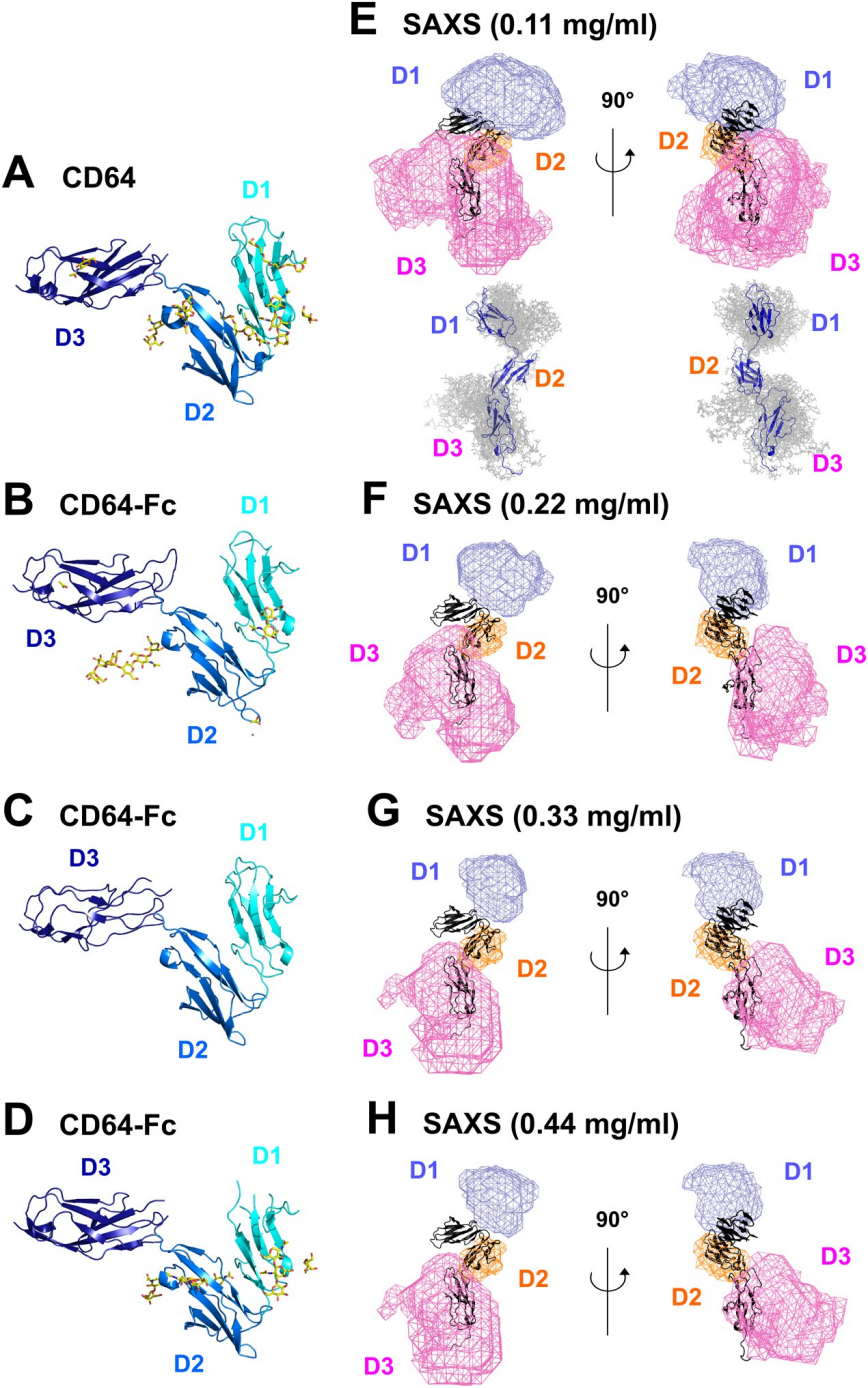
Comparison of the crystal structures of CD64 with density plots of the 100 best-fit models of CD64. (A-D) Ribbon diagrams of the four CD64 crystal structures correspond to unbound CD64 (PDB code 3RJD) and the three CD64-Fc co-crystals (PDB codes *B*, 4W4O, *C*, 4X4M and *D*, 4ZNE). The glycans in the crystal structures are shown as yellow sticks. (E-H) The density plots of the four sets of putative 100 best-fit CD64 models at four concentrations are shown. The energy-minimised starting structure of CD64 is shown as a black cartoon in the same views in all four panels. The 100 best-fit models were superimposed on the D2 domain. The volumes occupied by the D1, D2 and D3 domains in the 100 best-fit structures are represented as blue, orange and red wireframes respectively in each density plot. Two different views rotated by 90° of each density plot are displayed. In panel E, we also show a stick representation of 10 of the 100 best-fit CD64 models in a smaller scale to clarify how the density plots related to the atomistic models, with the best fit model shown as a blue ribbon.

Comparisons of the crystal structures and scattering models showed the arrangements of the D1 and D3 domains (Fig 7). The four CD64 crystal structures showed the “sea-horse” arrangement of the D1, D2 and D3 domains where the D1 and D2 domains were in proximity (Fig 7A–7D). By aligning the scattering best-fit models on the D2 domain (orange wireframe), the positions of the D1 and D3 domains were identified as mauve and pink wireframes respectively (Fig 7E–7H). These were compared with the energy minimised CD64 starting structure in black. As the CD64 concentration and amount of dimer increased, the D1 and D3 domains moved away from the D2 domain. The 0.11 mg/ml structure best-fitted the CD64 monomer structure seen in the four crystal structures.

**Fig 8:**
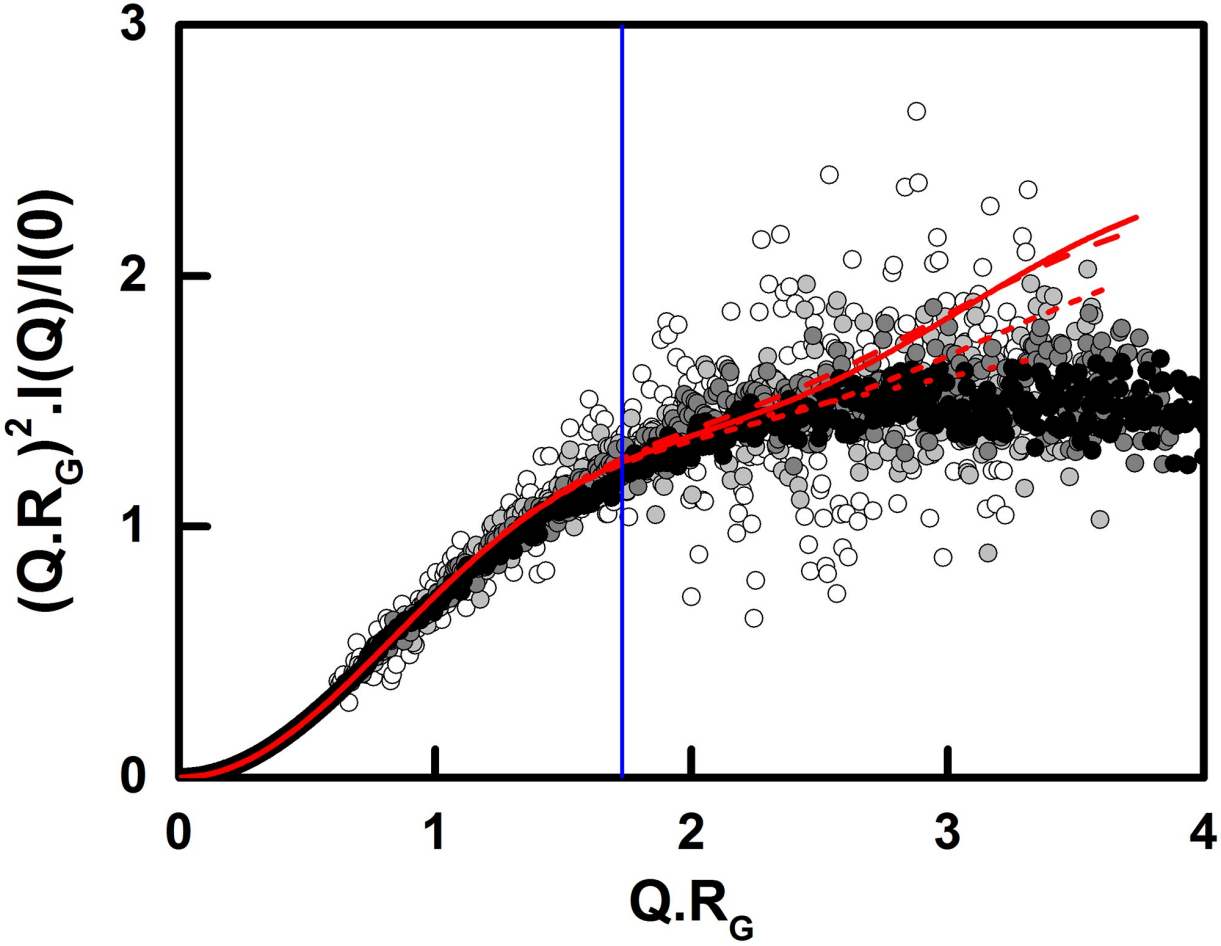
Experimental and modelled Kratky plots for CD64. The normalised dimensionless Kratky plots for 0.11, 0.22, 0.33 and 0.44 mg/ml CD64 are shown as white, grey, dark grey and black circles respectively. The Kratky plots for each of the modelled X-ray curves for each CD64 concentration are shown as dotted, short dash, long dash and solid red lines respectively. The blue vertical line represents the point of inflection at a *Q*.*R*_*G*_ value of 1.7.

Kratky plots provide information on protein flexibility in solution. Based on both the four experimental and four apparent best-fit curves, the Kratky plots using a *Q*-range up to 1 nm^-1^ showed that the eight curves all plateaued at an inflexion point of *Q*.*R*_*G*_ of 1.7 (Fig 8). Such a plateau is a signature of protein flexibility [43]. This plateau is independent of the concentration dependence seen in the Guinier analyses above (Fig 4C and 4D). Such an inflexion point indicated that unbound CD64 showed flexibility in its solution structure. The modelling of the 100 best-fit structures for the 0.11 mg/ml structure provided an estimate of this flexibility by showing a broader distribution of structures for the D3 domain compared to that for the D1 domain, when both were compared to the static D2 domain structure (Fig 7E). This observation predicted that CD64 flexibility mostly arises from the D2-D3 linker region, and some from the D1-D2 linker.

### Sedimentation coefficient modelling of CD64

The SAXS modelling was confirmed by AUC modelling. The experimental *s*^*0*^_20,*w*_ values were calculated using HYDROPRO for the four glycan-free crystal structures and the sets of 100 SAXS best-fit models (Fig 3E). The *s*^*0*^_20,*w*_ values were 2.33 S, 2.58 S, 2.41 S and 2.45 S for the PDB codes 3RJD, 4W4O, 4X4M and 4ZNE respectively. The averaged crystal structure *s*^*0*^_20,*w*_ value of 2.44 ± 0.10 S (dashed line, Fig 3E) thus agreed well with the experimental *s*^*0*^_20,*w*_ value of 2.53 ± 0.06 S CD64 at zero concentration. The agreement between experimental and modelled *s*^*0*^_20,*w*_ values is typically ±0.21 S [23]. In contrast, the averaged *s*^*0*^_20,*w*_ values for each of the four sets of 100 best-fit SAXS models were notably lower at 2.16 ± 0.01 S, 2.09 ± 0.01 S, 2.07 ± 0.01 S and 2.07 ± 0.01 S. This difference was attributed to the perturbations caused by dimers in the SAXS best-fit models, which caused the latter to become apparently more elongated and causing the observed reduction in the predicted *s*^*0*^_20,*w*_ values.

## Discussion

**Fig 9:**
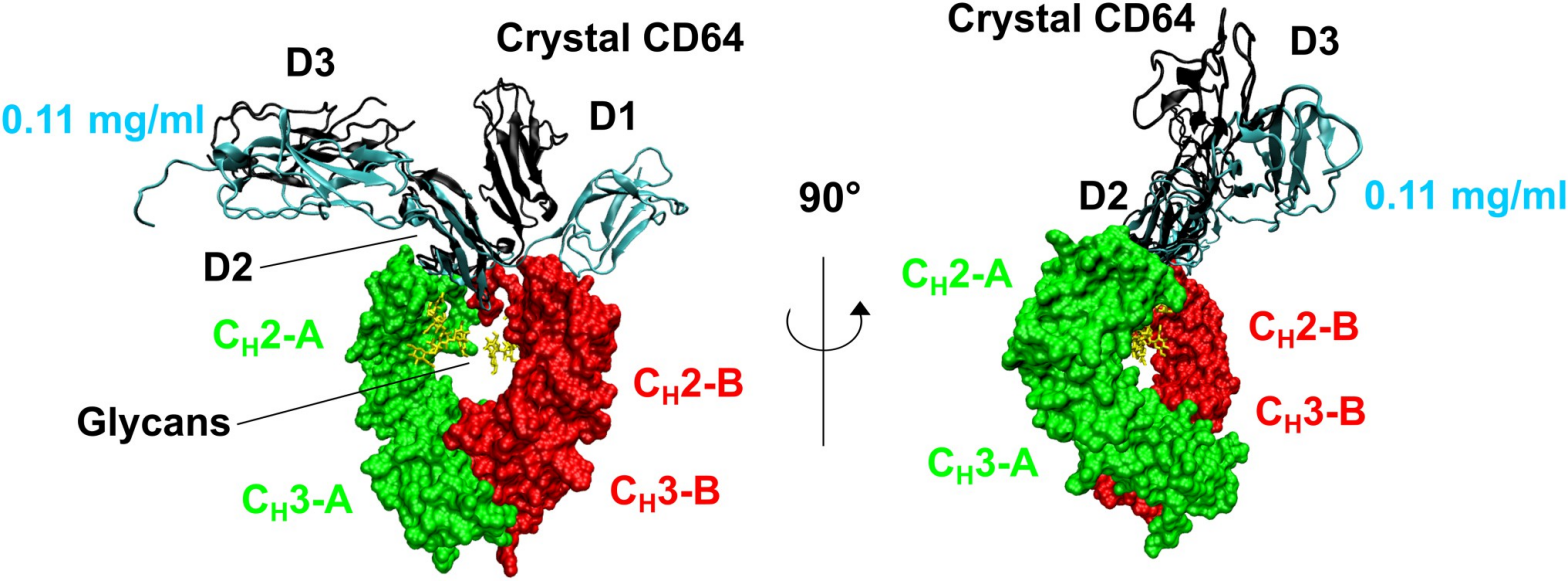
Superimposition of the CD64 best-fit models with the crystal structure of the Fc-CD64 complex. The best-fit ribbon model for 0.11 mg/ml CD64 was overlaid on top of the crystal structure of IgG1-Fc (green and red surfaces) in complex with CD64 (black cartoon) (PDB code 4X4M). The structures were aligned upon the D2 domain of CD64 and the two views were rotated about the vertical axis by 90°. The C_H_2 and C_H_3 represent the constant domains, and the—A or—B represent their chain identity in the crystal structure. The IgG1-Fc glycans found in the crystal structure are shown as yellow sticks. Note that the crystal CD64 structure corresponds to that of Fig 7C.

CD64 (FcγRI) exhibits distinct features compared to the other human FcγR receptors. It is the only receptor that bind monomeric IgG with high-affinity and possesses an extra extracellular domain D3. Here, despite potential issues caused by low concentrations and the presence of small CD64 dimer formation, we present the first solution structure for CD64 determined by AUC, SAXS and atomistic modelling. The presence of minor dimers is unlikely to be functionally significant, and may arise from the absence of glycosylation in the expressed protein. The best-fit solution structures consistently showed that CD64 retained most of if not all of the “sea-horse” structure seen crystallographically in the CD64-Fc complexes and free CD64. This D1-D2 angle was reported to be similar at 35° in the four crystal structures [12, 13, 16, 18]. The D1-D2 angle was stabilised by two interdomain salt bridges, three hydrogen bonds and hydrophobic interactions [13]. The free structure of the high affinity FcεRI receptor also shows a similar D1-D2 hinge angle as that for CD64. The crystal structures show that there is a substantial hydrophobic core packed in the D1-D2 hinge to stabilise the hinge angle, meaning that there would be an energetic cost in opening up the D1-D2 hinge. However, the crystal structures showed that the D2-D3 hinge angle was more variable, whereby a 19° difference was observed between the CD64-Fc complex and unbound CD64 [12, 18]. Unbound CD64 showed one salt bridge and four hydrogen bonds between the D2 and D3 domains [13]. In the modelling, the broadest distribution of CD64 structures was observed at the D2-D3 linker (Fig 9E). The position of the D3 domain appears to depend on its environment in the crystal lattice, its intrinsic mobility and the flexible linkers of CD64 [12, 18].

Our modelling outcome is summarised by superimposing the best-fit CD64 scattering structure at 0.11 mg/ml with the crystal structure of the Fc-CD64 complex (Fig 9). The D3 domain was located in the same position in both structures, which appears to be fortituous, while the D1 domain was slightly displaced between both structures. In this study, we have used a mutant CD64 expressed in bacteria. Because the mutant binds tightly to IgG1 Fc [16, 20], it appears unlikely that its mutations impact the CD64 conformation. Crystals for these four structures were grown in variable salt conditions, from 50 mM to 200 mM salt concentrations [12, 13, 16, 18]. It is not clear what contributed to the slightly more open D1-D2 conformation of CD64 deduced from SAXS data. The Kratky plot (Fig 8) demonstrated that the CD64 solution structure shows some inter-domain flexibility that is consistent with a slightly opened domain arrangement.

### Previous AUC and SAXS solution studies of CD64

The expression and purification of soluble recombinant CD64 with 19 mutations from *E*. *coli* was based on well-established protocols [16, 17, 19, 20]. This gave monomeric CD64, however AUC and SAXS all revealed dimer formation with increase in concentration. Interestingly SDS-PAGE of glycosylated CD64 from mammalian cells [53, 54] revealed higher molecular weight species which may be attributable to the presence of glycans or protein aggregates. These studies indicated that as much as 50% of CD64 oligomerised or aggregated [53–55]. These issues were challenging for data collection in the present AUC and SAXS study, however the use of concentrations well below 1 mg/ml CD64 where oligomers were minimal, together with a high intensity X-ray source to improve signal-noise ratios, enabled our study to be completed.

As far as is known, no *s*^*0*^_20,*w*_ values for CD64 by AUC have been reported to date. Here, the extrapolation of 42 data points for CD64 at low concentrations in two buffers to zero concentration permitted the *s*^*0*^_20,*w*_ value of 2.53 ± 0.06 S to be determined. This value is consistent with that of approximately 3 S for glycosylated human CD16A (FcγRIIIA) with two extracellular domains [54]. Human FcγRIIIB has been studied by AUC but no *s*^*0*^_20,*w*_ values were reported [56]. Recombinant murine FcγRII with two domains gave a *s* value of 2.5 S [57]. Thus our AUC results for CD64 are consistent with the literature. Likewise, as far as is known, CD64 has not been studied by SAXS previously. Human FcγRIIIB with two domains gave a *R*_*G*_ value of 1.88 nm by neutron small-angle scattering [21]. Our *R*_*G*_ values for CD64 at zero concentration were 3.28 nm and 3.42 nm (Fig 4C; Table 2), these being almost doubled compared to that for human FcγRIIIB.

### Atomistic modelling of CD64

A strategy for atomistic scattering curve modelling depends on the available data of interest. Both AUC and SAXS revealed a concentration dependence, attributable to low amounts of dimer formation. The impact of these dimers was successfully managed in the atomistic modelling by individually fitting each of the four SAXS curves at 0.11–0.44 mg/ml to identify their effect on the apparent solution structure of CD64. The poorer signal-noise ratios at lower concentrations prevented a satisfactory extrapolation of the four curves to zero concentration. Accordingly we performed 12 separate fit analyses at four concentrations based on a library of 279,162 trial structures (Table 2). From Table 2, only two experimental curves at 0.22 mg/ml, E10 and D10, gave rise to 6,980 and 8,100 best-fit models respectively with *R-factors* of ≤ 5%. However most of the other scattering curves gave *R-factors* of ≤ 10%. The 12 sets of 100 best-fit models were examined to see if any of the 100 best-fit models fitted more than one experimental X-ray scattering curve. Interestingly, 57 models fitted six out of the 12 experimental curves, hence indicating the reproducibility of the modelling fits, even though the scattering data at 0.11 mg/ml was noisy. The resulting density plots of the 100 best-fit models showed that only a limited number of conformations were allowed (Fig 7E–7H). That at the lowest concentration corresponded closely to the CD64 crystal structure (Fig 7E), and was concluded to be the best outcome.

The Kratky plots investigated potential flexibility in the CD64 solution structure. In these, globular compact proteins display a clear parabolic peak at *Q*.*R*_*G*_ = √3 (or 1.73), giving rise to a bell-shaped curve. An ideal Gaussian chain has a 1/*Q*^2^ dependence of *I*(*Q*) and therefore presents a plateau at large *Q* values. An extended thin chain with negligible thickness presents a plateau over a specific range of *Q*, followed by a monotonic increase, which usually corresponds to unfolded disordered proteins. Proteins with more than two globular regions connected by intrinsically disordered linkers present dual folded and non-folded behaviour [42]. The D1, D2 and D3 domains in CD64 were potentially joined by two flexible linkers, preceded and followed by disordered N-terminal and C-terminal tails. The observation of a plateau in the Kratky plots with a point of inflexion at *Q*.*R*_*G*_ = 1.73 (red lines, Fig 8) indicated flexibility in CD64 because this is intermediate between the two extremes of a globular folded protein and a disordered protein (circles, Fig 8) [43, 58–60].

### CD64 interaction with human IgG

The order of the binding strength of CD64 to monomeric IgG is IgG1 = IgG3 > IgG4 [1, 2, 16, 17, 20]. IgG2 shows no detectable binding to CD64. The IgG3 and IgG4 subclasses also exhibit high-affinity binding to CD64 and have the same lower hinge sequence as IgG1, thus CD64 is expected to show very similar if not identical interactions with IgG3 and IgG4 as for IgG1. The position of the D1, D2 and D3 domains will potentially influence the interaction with IgG1-Fc [12]. The best-fit solution models at 0.11 mg/ml CD64 (cyan) explain the CD64-Fc interaction. In this, neither the D1 or D3 domains sterically clashed with the C_H_2 domain (Fig 9). Thus the CD64 interaction with the Fc region is facilitated by the non-covalent interactions between D1 and D2 to maintain the high-affinity interaction. Interestingly, it has been reported that only the D2 domain is responsible for this interaction [18], i.e. a construct with D1 from FcγRIIIA and D2 from CD64 showed binding for IgG1 identical to that of the CD64 D1 and D2 domains. The D3 domain in the best-fit models may exhibit a range of conformations, as flexibility in the D2-D3 linker (suggested by the Kratky plots) would facilitate the CD64 interaction with full-length IgG, because CD64 is tethered to the membrane (Fig 1A). CD64 is bound to monomeric IgG with a short half-life of 3 min, thus the high-affinity CD64 receptor should be occupied *in vivo* [2]. Interestingly the D3 domain was also the most disordered in the crystal structures with several D3 residues not being resolved [12, 18].

### CD64 glycosylation and therapeutic development

In native CD64, the D1, D2 and D3 domains contain two, three and two glycosylation sites respectively (Fig 1A). Therefore CD64 is the most heavily glycosylated FcγR receptor, with 30% of its mass being glycans [61]. Crystallography revealed parts of the glycan (Fig 7A–7D), namely six in unbound CD64 [13], five in one CD64-Fc complex [18], and two in the other [12]. Four of the mutations in our recombinant CD64 alter an asparagine residue which would otherwise be a glycosylation site (Fig 1C). CD64 in our study was expressed in *E*. *coli* and has no glycan chains. Glycans on CD64 result in an increased affinity for human IgG^16^ compared to non-glycosylated CD64, although others have reported negligible differences in affinities [12, 20]. The crystal structures of CD64-Fc do not show that CD64 glycosylation was involved with IgG complex formation [16]. Nonetheless it is possible that the seven glycan chains may stabilise the compact seahorse structure in solution [12, 13, 16, 18].

CD64 is a valid target for drug development given the role of Fc receptors in autoimmune diseases [15, 62]. CD64 is upregulated in inflammatory diseases such as arthritis, systemic lupus erythematosus and inflammatory bowel disease [6, 8]. Other human FcγR subclasses have genetic variation and these result in differences in clinical association and impact [63]. Recombinant CD64 might be effective in treating arthritis in humans as it has been shown to have a therapeutic anti-inflammatory effect in mice with arthritis [64, 65]. We speculate that the identification of flexibility in the D1-D2 and D2-D3 linkers in CD64 may mean that compounds that bind to the open D1-D2 and D2-D3 conformations could be designed to modify their binding to IgG in order to suppress CD64 upregulation in disease.

## Conclusion

In summary, we have studied the solution structure of the IgG high affinity receptor CD64 by combining analytical ultracentrifugation, X-ray and neutron scattering, and molecular dynamics simulations. By working at the lowest concentrations, we determined the solution structural parameters for CD64. Detailed Monte Carlo simulations provided a full range of trial structures for the three domains of CD64. The best fits of these structures to the scattering curves showed that the CD64 solution structure resembled its crystal structure, but identified previously unrecognised flexibility between the D1, D2 and D3 domains. This leads us to suggest that flexibility forms a key part of the function of CD64 and facilitates the high affinity binding of IgG1 to its receptor.

## Supporting information

S1 Fig The original SDS-PAGE image used for Fig 2B in the main text is shown. Lanes 8, 9 and 10 (protein markers, non-reduced CD64 and reduced CD64) were used in Fig 2B.
(JPG)

S1 Table Supporting information is presented that summarises the SAXS sample details, data collection, analysis, and 3D modelling details for CD64. (DOCX)

S1 File The supporting information zip file provides the 100 best-fit models for CD64 corresponding to the fit searches of the X-ray curve at 0.11 mg/ml. (ZIP)

## Abbreviations

AUC: analytical ultracentrifugation
CCP-SAS: collaborative computational project for small angle scattering
CD64: cluster of differentiation 64
D1, D2 and D3: the three domains of CD64
FcγR: Fc gamma receptor
IgG: immunoglobulin G
SAXS: small angle X-ray scattering
