# Supplementary figures and images for: The solution structure of the unbound IgG Fc receptor CD64 resembles its crystal structure: Implications for function
Source: PLoS One. 2023 Sep 21;18(9):e0288351. doi: 10.1371/journal.pone.0288351 (PMC10513344; doi:10.1371/journal.pone.0288351)

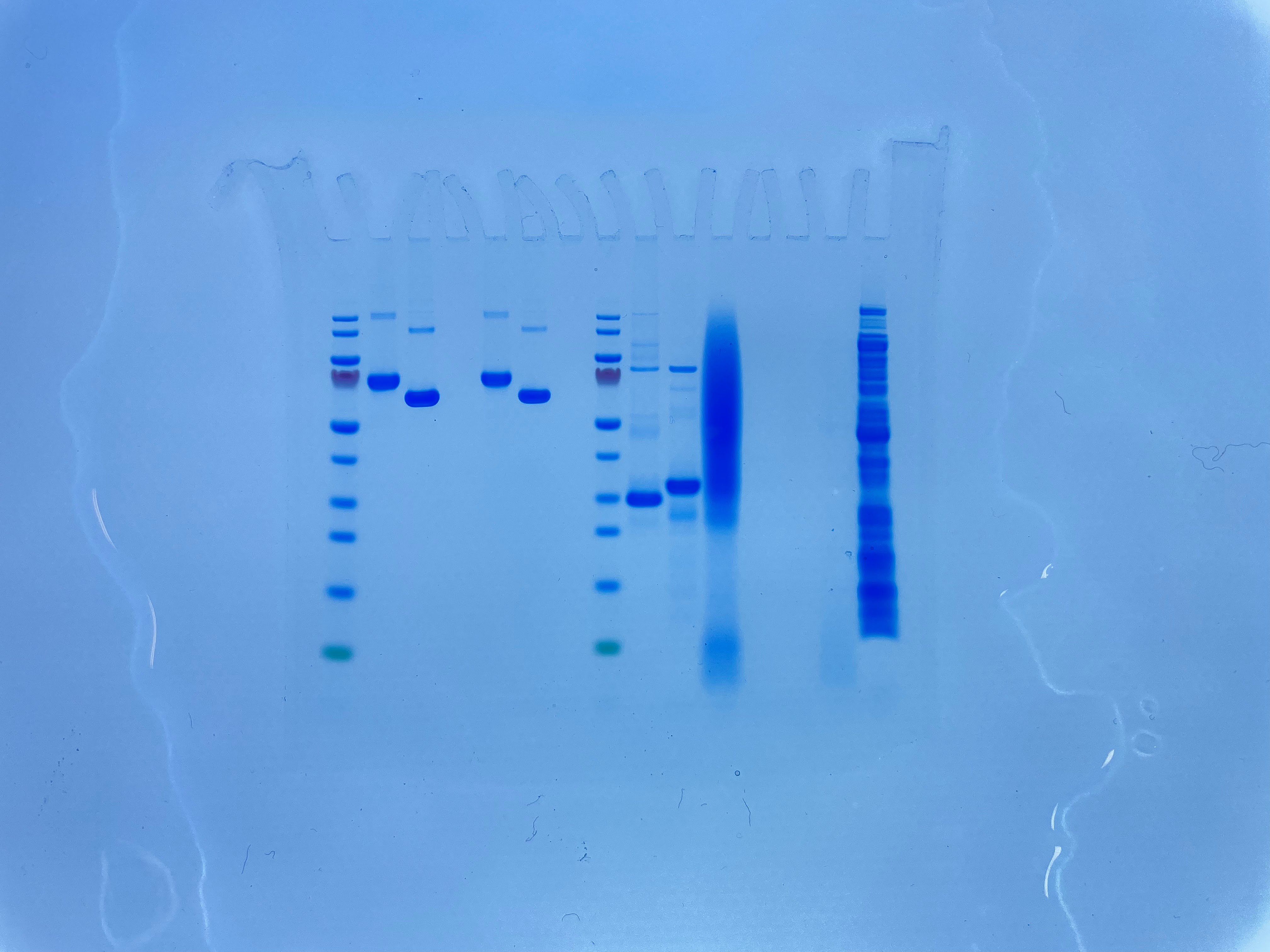

Supplement: S1 Fig — Lanes 8, 9 and 10 (protein markers, non-reduced CD64 and reduced CD64) were used in Fig 2B. (JPG) [file pone.0288351.s001.jpg]
