# Supplementary material for: The solution structure of the unbound IgG Fc receptor CD64 resembles its crystal structure: Implications for function
Source: PLoS One. 2023 Sep 21;18(9):e0288351. doi: 10.1371/journal.pone.0288351 (PMC10513344; doi:10.1371/journal.pone.0288351)
Supplement: S1 Table — (DOCX) [file pone.0288351.s002.docx]

**Supporting Information:** **SAXS sample details, data collection, analysis, and 3D modelling details for CD64.**

| (*a*) Sample details | | | |  |
| --- | --- | --- | --- | --- |
| Organism |  |  |  |  |
| Source (Catalogue No. or reference) | Uniprot Accession Code P12314, FCGR1_HUMAN |  |  |  |
|  | Sample 1 | Sample 2 | Sample 3 | Sample 4, *etc* |
| *Scattering particle composition* |  |  |  |  |
| Protein(s)^a^ | 100% |  |  |  |
| DNA/RNA(s)^b^ | 0% |  |  |  |
| Carbohydrates/glycans^c^ | 0% |  |  |  |
| Stoichiometry of components | 1:0:0 |  |  |  |
| *Sample environment/configuration* |  |  |  |  |
| Solvent composition^d^ | 30 mM Tris, 150 mM NaCl, pH 7.6 |  |  |  |
| Sample temperature (°C) | 20°C |  |  |  |
| In beam sample cell^e^ | Capillary |  |  |  |
| *Batch measurements* |  |  |  |  |
| Sample concentration(s), mg/ml or g/cm^3^ | 0.11 mg/ml | 0.22 mg/ml | 0.33 mg/ml | 0.44 mg/ml |
| *Size Exclusion Chromatography SEC-SAS* |  |  |  |  |
| Sample injection concentration, mg/ml or g/cm^3^ | Not used |  |  |  |
| Sample injection volume, mL |  |  |  |  |
| SEC column type |  |  |  |  |
| SEC flowrate, mL/min |  |  |  |  |
| (*b*) SAS data collection | | | |  |
| Data acquisition/reduction software | ScÅtter | | |  |
| Source/instrument description or reference | Diamond B21 | | |  |
| Measured *q*-range (*q_min_* – *q_max_*; Å^-1^, nm^-1^) | 0.032-3.8 nm^-1^ | | |  |
| Method for scaling intensities^f^ |  | | |  |
| Exposure time(s), number of exposures. For SEC-SAS, final number of sample frames used for averaging. | 1 sec, 30 frames | | |  |
| Additional relevant details^g^ |  | | |  |
| (*c*) SAS-derived structural parameters | | | |  |
| Methods/Software |  |  |  |  |
| *Guinier Analysis* | Sample 1 | Sample 2 | Sample 3 | Sample 4, *etc* |
| *I*(0) ± σ (cm^-1;^ a.u) | 0.0244 ± 0.0009 | 0.0286 ± 0.0016 | 0.0308 ± 0.0025 | 0.0373 ± 0.0013 |
| *R*_g_ ± σ (Å, nm) | 3.44 ± 0.28 nm | 3.67 ± 0.13 nm | 3.86 ± 0.07 nm | 4.02 ± 0.04 nm |
| *min < qR_g_* < *max* limit (or data point range) | 0.5-1.6 | 0.5-1.6 | 0.5-1.6 | 0.5-1.6 |
| Linear fit assessment (definition)^h^ |  |  |  |  |
| *PDDF/P(r) analysis* | Sample 1 | Sample 2 | Sample 3 | Sample 4, *etc* |
| *I*(0) ± σ (cm^-1;^ a.u.) |  |  |  |  |
| *R*_g_  ± σ (Å, nm) | 3.56 ± 0.13 nm | 3.70 ± 0.06 nm | 3.79 ± 0.05 nm | 3.95 ± 0.03 nm |
| *d*_max_ (Å, nm) | 12 nm | 12 nm | 12 nm | 12 nm |
| *q*-range (Å^-1^, nm^-1^) | 0.032-3.8 nm^-1^ |  |  |  |
| *P*(*r*) fit assessment (definition)^i^ |  |  |  |  |
| (*d*) Scattering particle size | | | |  |
| Methods/Software |  |  |  |  |
|  | Sample 1 | Sample 2 | Sample 3 | Sample 4, *etc* |
| *Volume estimates* |  |  |  |  |
| Porod volume, *V_p_* (Å^3^, nm^3^) |  |  |  |  |
| *Molecular weight (M) estimates (kDa)* |  |  |  |  |
| From chemical composition | 32,012 |  |  |  |
| From SAS, concentration independent method^j^ |  |  |  |  |
| From *I*(0)/concentration^k^ |  |  |  |  |
| Partial specific volume, ν (cm^3^/g) | 0.7363 ml/g |  |  |  |
| Contrast, Δ*ρ* (cm^-2^) |  |  |  |  |
| From SAS-independent measure^l^ (method) | 32,000 (mass spectrometry) |  |  |  |
| (*e*) Modelling (a complete sub-panel for each method) | | | |  |
| *Shape modelling method(s) (if used)* |  |  |  |  |
|  | Sample 1 | Sample 2 | Sample 3 | Sample 4, *etc* |
| Software | Not used |  |  |  |
| *q-*range for fit (*q_min_* – *q_max_*; Å^-1^, nm^-1^) |  |  |  |  |
| Symmetry/anisotropy assumptions |  |  |  |  |
| Number of individual model reconstructions |  |  |  |  |
| *χ*^2^, CorMap *P*-values for fit |  |  |  |  |
| For multiple phase models: *R*_g_ values (Å, nm) and relative phase volumes (Å^3^, nm^3^) |  |  |  |  |
| *Atomistic modelling methods (if used)* |  |  |  |  |
|  | Sample 1 | Sample 2 | Sample 3*.* | Sample 4, *etc* |
| Software | CCP-SAS and SASSIE |  |  |  |
| *q-*range for fit (*q_min_* – *q_max_*; Å^-1^, nm^-1^) | 0 to 1.00113 nm^-1^ |  |  |  |
| Symmetry/anisotropy assumptions | None |  |  |  |
| Number of individual model reconstructions | 279,162 |  |  |  |
| *χ*^2^, CorMap *P*-values for fit | R-factor 7.54% |  |  |  |
| (*f*) Data and model deposition |  |  |  |  |
|  | Sample 1 | Sample 2 | Sample 3 | Sample 4, *etc* |
| SASBDB IDs | SASDP89 |  |  |  |

^a^ Recommended description is UniProt ID (<https://www.uniprot.org/>), including the recommended UniProt name with the amino acid sequence range of the construct measured by SAS, plus any tags, post-translational modifications, ligands, cofactors, metals, etc. If UniProt ID’s are not available the recommendation is to quote the NCBI accession and protein name (<https://www.ncbi.nlm.nih.gov/guide/proteins/>). If a sequence has neither UniProt nor NCBI identifiers, or if the description is too long for the table format, provide an abbreviated title with a reference to the location where exact sequences with modifications, etc., can be found.

^b^ If possible, quote the relevant GenBank (<https://www.ncbi.nlm.nih.gov/genbank/>), RNACentral (<https://rnacentral.org/>) or ENA accession number (<https://www.ebi.ac.uk/ena/browser/home>), specifying any modifications, derivatives, *etc*. If the description is too long for the table format, provide an abbreviated title with a reference to where the exact sequence with modifications, *etc.*, can be found.

^c^ If possible, quote the GlyTouCan (<https://glytoucan.org/>) accession code, or information from GlyGen (<https://www.glygen.org/> ). For chemical groups, use standard nomenclature, *e.g.*, for glycans, it is recommended to adhere to the Symbol Nomenclature for Glycans (SNFG) protocols (<https://pubmed.ncbi.nlm.nih.gov/31184695/>) and /or IUPAC nomenclature (<https://iupac.org/what-we-do/nomenclature/>).

^d^ Provide complete solvent description (including buffer with pH, salts and any additives, *e.g.*, free radical scavengers).

^e^ *e.g.*, cell type, pathlength, flow cell, coflow, etc.

^f^ Strongly recommend absolute scaling of the scattering intensities, cm^-1^, with reference to a standard, otherwise specify relative or arbitrary units (a.u.).

^g^ *e.g.*, data smearing/desmearing, data merging, data re-binning, data normalization, standard experimental errors or otherwise, *etc*. For SANS recommend wavelength λ, Δλ/λ, sample-to-detector distances, source/sample to aperture distances, and collimation distances.

^h^ *e.g.*, linear correlation coefficient.

^i^ Recommend reciprocal space fit to experimental data (χ^2^; CorMap *P*).

^j^ *e.g.*, estimated from *V_p_* and knowledge of partial specific volume and hydration (Trewhella *et al.*, 2017), or using volume of correlation *V_c_* (Rambo & Tainer, 2013), SAXSMow (Piiadov *et al.*, 2019), or DatBayes (Hajizadeh *et al.*, 2018) from the ATSAS suite (Manalastas-Cantos *et al.*, 2021).

^k^ Either from equation 1 (Trewhella *et al.*, 2017) or relative to a standard.

^l^ *e.g.*, Multiple Angle Laser Light Scattering (MALLS), Analytical Ultra-Centrifugation (AUC), *etc*.
